# Evaluation of the Toxic Effect of *Bauhinia purpurea* Mediated Synthesized Silver Nanoparticles against *In-vitro* and *In-vivo* Models

**DOI:** 10.3390/toxics11010009

**Published:** 2022-12-22

**Authors:** Nagarajan Shobana, Pandurangan Prakash, Antony V. Samrot, Subramanian Saigeetha, Mahendran Sathiyasree, Rajendran Thirugnanasambandam, Sridevi Visvanathan, Basanta Kumar Mohanty, Gokul Shankar Sabesan, Shanmugaboopathi Dhiva, Rajan Renuka Remya, Senthilkumar Pachiyappan, Samraj Wilson

**Affiliations:** 1 Department of Biotechnology, School of Bio and Chemical Engineering, Sathyabama Institute of Science and Technology, Chennai 600119, Tamil Nadu, India; 2 School of Bioscience, Faculty of Medicine, Bioscience and Nursing, MAHSA University, Jenjarom 42610, Selangor, Malaysia; 3 Department of Biotechnology, School of Biosciences and Technology, Vellore Institute of Technology, Vellore 632014, Tamil Nadu, India; 4 Centre for Ocean Research (DST—FIST Sponsored Centre), MoES—Earth Science & Technology Cell, Sathyabama Institute of Science and Technology, Chennai 600119, Tamil Nadu, India; 5 Unit of Biochemistry, Faculty of Medicine, AIMST University, Semeling, Bedong 08100, Kedah Darul Aman, Malaysia; 6 Faculty of Medicine, Manipal University College Malaysia (MUCM), Jalan Padang Jambu, Bukit Baru 75150, Melaka, Malaysia; 7 Department of Microbiology, Sree Narayana College, Alathur, Palakkad 678682, Kerala, India; 8 Department of Biotechnology, Bharath Institute of Higher Education and Research, Bharath University, Chennai 600073, Tamil Nadu, India; 9 Department of Chemical Engineering, Saveetha Engineering College, Thandalam, Chennai 602105, Tamil Nadu, India; 10 Department of Botany, St. John’s College, Tirunelveli 627002, Tamil Nadu, India

**Keywords:** metal nanoparticles, nanotoxicity, plant gum

## Abstract

Metal nanoparticles, such as gold nanoparticles, silver nanoparticles, etc., have many benefits and have been in use for a very long time. Nevertheless, a number of concerns have been raised about the environmental impact and the possibility of exposure to various living systems at the moment. Thus, in this study, silver nanoparticles were synthesized by using plant gum from *Bauhinia purpurea* and characterization was done using UV—Visible Spectroscopy, Scanning Electron Microscopy, X—ray Diffraction, etc. To determine the accumulation and toxic effects caused by the nanoparticles, *Eudrilus eugeniae*, *Danio rerio*, and their embryos were exposed to the synthesized silver nanoparticles and evaluated using microscopic observation, histology, and Inductively Coupled Plasma Optical Emission Spectrometry (ICP—OES).

## 1. Introduction

Nanotechnology has emerged as one of the most promising technologies in a variety of fields. Metal nanoparticles have higher electrical conductivity, roughness, and can strengthen metals and alloys which can be used in fields such as medicinal, agricultural, environmental, and physiochemical [1,2]. The antiviral or antibacterial effect of nanoparticles is influenced by their size, capping agent, and composition [3]. The large surface area and smaller particle size of the nanoparticles allow them to be employed in the techniques used for detecting viral infection, such RT—PCR, ELISA, and RT—LAMP [4]. Chemical composition and reaction conditions (e.g., temperature and pH) can be adjusted to change the morphological features of the nanoparticles (such as size and shape) [5]. However, synthesized nanomaterials may encounter some challenges when applied to specific applications, such as stability in hostile environments, lack of understanding of fundamental mechanisms, bioaccumulation/toxicity, need for extensive analysis, need for skilled operators, problem sizing [6]. There have been numerous applications of silver nanoparticles, such as antibacterial, anticancer, anti—inflammatory, and wound—care [7,8,9,10,11]. Due to the remarkable reactivities of silver nanoparticles, as well as the greater surface—to—volume ratios, they have opened new areas in biomedical research [2,9].

The most common approach for synthesizing silver nanoparticles is chemical synthesis, which employs harsh and toxic chemicals to reduce silver ions and stabilize nanoparticles [12]. The biogenic synthesis approach has become more popular as the chemical method requires the usage of chemicals and reagents which are hazardous and can cause health and environmental problems [13,14]. Biogenic nanoparticles can be synthesized by using the by-products of an organism’s metabolism as a reducing and stabilizing agent, such as bacteria, fungi, and plants [15]. It helps in decreasing the toxicity, increases stability, and enhances physiochemical properties [16]. Polyoxometalates with mixed valence, polysaccharides, Tollen’s compounds, biological preparations, and irradiation methods are some of the green synthesis approaches. They differ from conventional methods of involving toxic chemicals [16]. Cost—effectiveness, environmental friendliness, and the potential to synthesize nanoparticles that are more compatible to pharmaceuticals, medicinal, agronomical, and environmental applications are all advantages of green nanoparticle synthesis over physical and chemical approach [17]. In this study, silver nanoparticles have been synthesized by using the plant gum of *Bauhinia purpurea*, commonly called purple orchid, butterfly tree, orchid tree, camel’s foot, etc., as the reducing agent [18]. The synthesized nanoparticles were characterized both spectrophotometrically and microscopically. The toxicity of the nanoparticles was checked against animal models such as earthworm, zebrafish, and its embryos. Since many of their genes and critical pathways are so similar to those of humans, they are recognized as important markers for toxicity. Earthworms are ideal and easily adjust to new surroundings and are good candidates for assessing metal toxicity due to the reduced cost, accessibility, epigenetics, and rapid reproduction. The entire genome of the zebrafish has been sequenced, making it a great resource for toxicity research [19].

## 2. Materials and Methods

### 2.1. Materials Used

All methods were performed in accordance with the relevant guidelines and regulations. Crude gum of *Bauhinia purpurea* was collected in the summer from Bengaluru, India. An incision in the tree’s bark was created, and gum exudate was collected repeatedly. The gum was shade dried before being pulverized using a mortar and pestle and stored in an airtight container [20]. All the chemicals used in this study were of analytical grade.

### 2.2. Synthesis of Silver Nanoparticle using Bauhinia purpurea Gum

*Bauhinia purpurea* gum was chosen as the bio-reducing agent for the fabrication of silver nanoparticles. First, 0.5 g of powdered gum was dispersed in 100 mL of distilled water and heated at 60 °C for 3 to 4 h with constant stirring. The solution was cooled after being filtered through muslin fabric with a pore size of 2 mm. Then, 100 mL of the gum solution was mixed with 1 mM silver nitrate (AgNO_3_) and left undisturbed for 24 h in the dark. After 24 h, the obtained nanoparticles were centrifuged at 10,000 rpm, pellets were collected, washed thrice using distilled water, and then lyophilized [21].

### 2.3. Bio-Physical Characterization of Silver Nanoparticles

The silver nanoparticle formation was examined by UV–Vis spectrophotometer (Shimadzu, UV 3600 PLUS, Kyoto, Japan). The Fourier—Transform Infrared Spectroscopy (FT—IR) measurements were done using Shimadzu, IRTRACER 100, Kyoto, Japan. Scanning Electron Microscopy (SEM) and elemental analysis was performed by using Zeiss Ultra Plus, Oberkochen, Germany. The shape of the nanoparticles was analyzed using transmission electron microscopy (TEM) (FEI, Titan Themis 300 kV, Hillsboro, OR, USA). The X—Ray powder Diffraction (XRD) spectra were recorded (PANalytical Corporation, X’Pert, Almelo, Netherlands) along with its stability via Zeta Potential analysis (ZETASIZER Nano Series ZSP; Malvern Instruments, Worcestershire, UK).

### 2.4. Collection, Maintenance, and Pre-Treatment of Earthworms and Zebrafishes

*Eudrilus eugeniae* were purchased from SS Vermicompost, Chennai. Acclimatization to laboratory conditions was done for 5 to 6 days prior to experiments. Cow dung (dried one) was given as a feed once a day and sprinkled with water each day. Four—month—old adult male zebrafishes (*Danio rerio*) were obtained from Tarun fish farm, Chennai and housed in glass aquariums with adequate aeration. Before the introduction of silver nanoparticles, they were given 3 to 4 days to adjust to the surroundings. It was fed twice a day with Daphnia eggs and Tubifex worms and had its water changed once a week [22].

### 2.5. Breeding, Spawning and Maintenance of Zebrafishes Embryos

Zebrafishes of both sexes were placed into a breeding tank and were allowed to mate at a ratio of 2:1 (male:female). Spawning and fertilization took place within 30 min and the fertilized eggs were collected, cleaned, and kept in E3 medium (sodium chloride, calcium chloride, magnesium chloride, potassium chloride; pH 7.2) which were then transferred to a 6-well plate containing the same medium (20 eggs/plate) using a Pasteur pipette [23].

### 2.6. Exposure to Silver Nanoparticles

Ten earthworms were transferred into large glass beakers, which contained silver nanoparticles (10 µg and 20 µg concentrations), which were dispersed in 10 mL of distilled water. After 1 h, the earthworms were transferred to their holding containers after interacting with silver nanoparticles. Similarly, 10 fishes were placed in glass tanks containing different concentrations of silver nanoparticles. Fishes were fed regularly as mentioned in the aforementioned section; the study lasted for 30 days. Two sets/treatment groups were maintained for each set of animals. Histology experiments were done on one set, while the population study for earthworms and adaptability studies for zebrafishes were done with the other set.

The toxicity study on zebrafish eggs was carried out as follows: Recovered zebrafish eggs were treated with different concentrations of silver nanoparticles and incubated at 28.5 °C in triplicates with the same concentrations of silver nanoparticles. The embryogenesis was observed using a Stereozoom Microscope (Leica M205A, Leica Microsystems, Stockach, Germany) starting at the time of nanoparticle exposure and was tracked over time [24].

### 2.7. Observation of Phenotypical Changes

For all the species, phenotypic changes such as color change, behavioral change, and death were observed, and cumulative data were recorded. On the embryos, apical observations and embryonic abnormalities, such as coagulation of embryos, absence of somite development, and lack of heartbeat, were perceived in addition to hatching.

### 2.8. Histopathological Studies and ICP–OES

On the 10th and 20th days after exposure, animals from each concentration were euthanized for histological investigations. Earthworm’s foreguts, midguts, and hindguts, as well as zebrafish eyes, gills, livers, and intestine were dissected and fixed in a 10% formaldehyde solution. After being embedded in wax, dehydrated with ethanol, and sectioned with a microtome, the tissues were sectioned and stained with Hematoxylin and Eosin (H&E). A fully automated Inverted Microscope was used to examine the samples (Leica DMI6000 B, Leica Microsystems, Stockach, Germany). The fishes and earthworms were collected and dissolved into a solution containing 5 mL of conc. nitric acid (HNO₃) and then with 5 mL conc. hydrofluoric acid (HCl). The solution was subjected to microwaving. The metal deposition in the solution was measured by Inductively Coupled Plasma Atomic Emission Spectroscopy (ICP–OES) [12,25].

## 3. Results

### 3.1. Characterization of Synthesized Silver Nanoparticles

**Figure 1:**
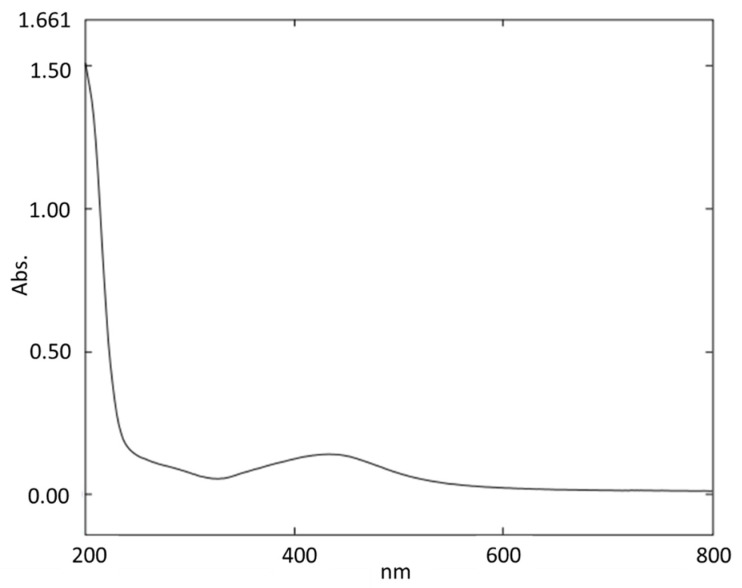
UV–Vis spectroscopy for the synthesized silver nanoparticles.

The absorption band at 450 nm indicated the formation of silver nanoparticles mediated by *Bauhinia purpurea* gum (Figure 1). Samrot et al. [13] produced silver nanoparticles using gum of *Araucaria heterophylla*, where the absorbance peaks of the silver nanoparticles were seen between 420 nm and 450 nm.

**Figure 2:**
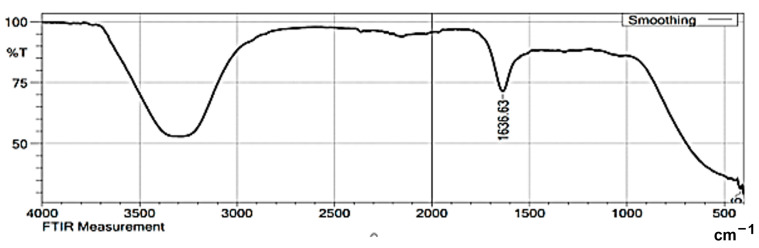
FTIR spectroscopy for the synthesized silver nanoparticles.

**Figure 3:**
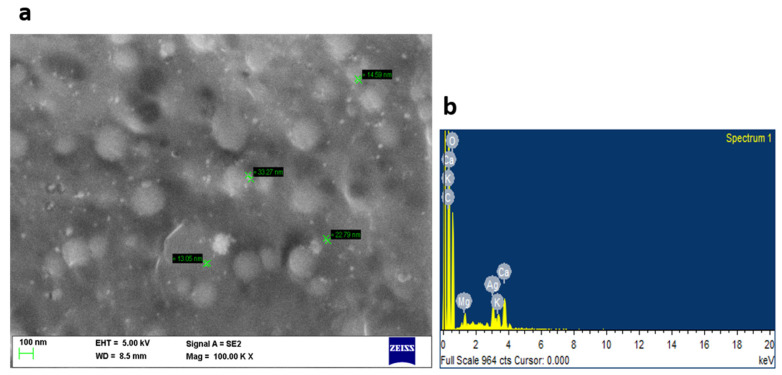
(**a**) SEM analysis, (**b**) EDX analysis for the synthesized silver nanoparticles.

**Figure 4:**
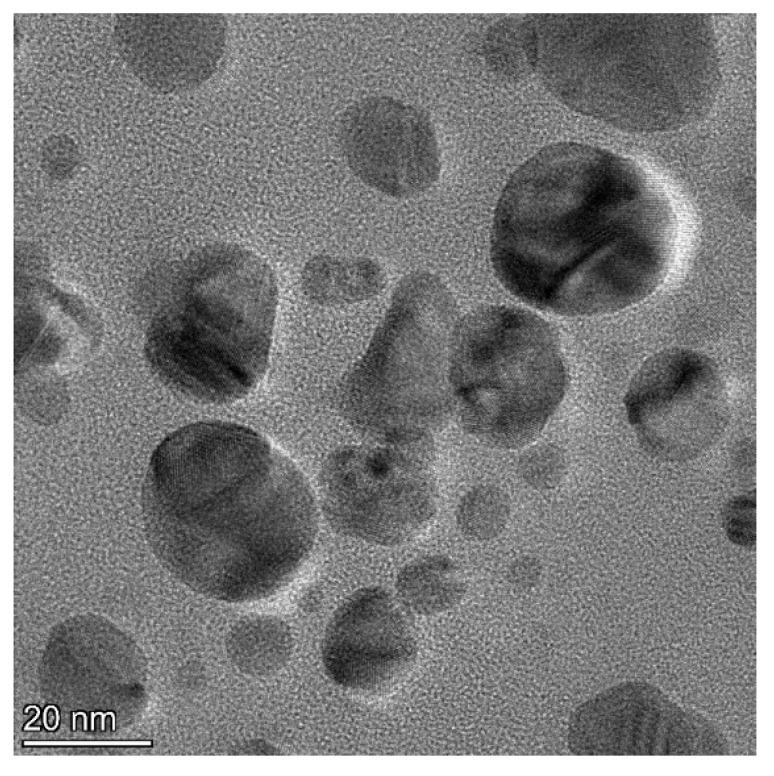
TEM analysis for the synthesized silver nanoparticles.

**Figure 5:**
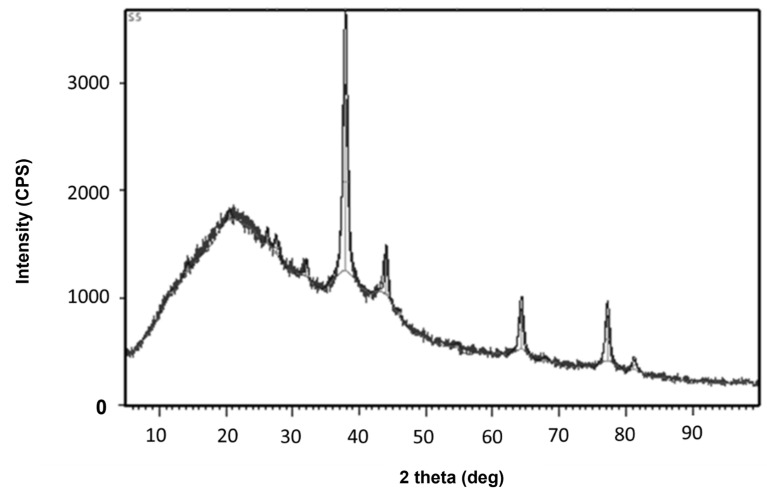
XRD analysis for the synthesized silver nanoparticles.

**Figure 6:**
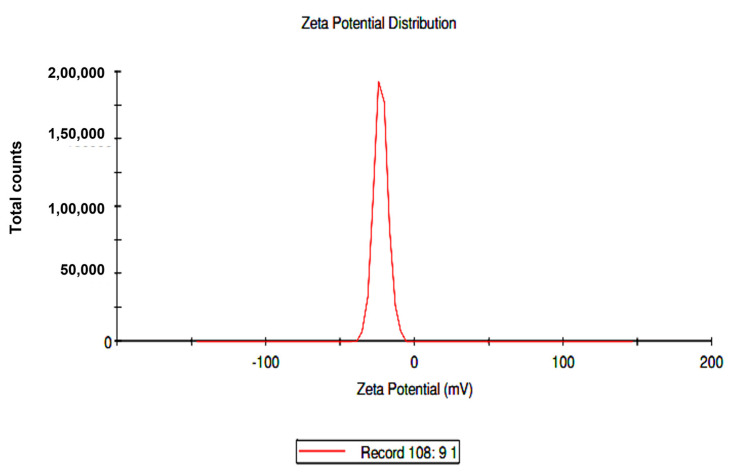
Zeta Potential analysis for the synthesized silver nanoparticles.

The biomolecules present in the gum solution help in capping and stabilizing the nanoparticles which were determined using Fourier Transform Infrared Spectroscopy (FTIR) studies (Figure 2). O–H stretching in the alcohols and phenols were confirmed by the prominent peak at the wavelength position at 3000–3500 cm^−1^. Peaks at 2400 cm^−1^ indicated carbonyl bond group (O=C=O) stretching. The peak at 1636 cm^−1^ is the strong indicator of alkenes (C=C) stretching. The Scanning Electron Microscopy (SEM) image shows a dense population of spherical, or nearly spherical, shaped nanoparticles with sizes ranging from 13 to 20 nm (Figure 3a). As previously reported, the Energy Dispersive X-Ray (EDX) spectrum (Figure 3b) showed silver with a significant signal peak at ∼3 KeV [22,26]. The silver nanoparticles synthesized using the aqueous extracts of three different stages of ripened *Capsicum annum* (green, yellow, red) ranged between 30 and 80 nm, which was further confirmed by Atomic Force Microscopic images [9]. Further, the shape of the synthesized silver nanoparticles was analyzed using Transmission Electron Microscopy (TEM) analysis (Figure 4), which correlated with the SEM result. The surface morphology of silver nanoparticles synthesized using aqueous extract of fresh leaves of *Pedalium murex* showed even shape and spherical shaped nanoparticles [27]. X-Ray Diffraction (XRD) analysis showed the peaks of 2θ at 38°, 43°, 65°, and 78° matching the JCPDS (File no.: 89—3722), which denoted that it had FCC structure [27] (Figure 5). Silver nanoparticles prepared using ethanolic extract of fruits of *Santalum album* were crystalline and had a face centered cubic crystal structure [28]. The Zeta Potential analysis revealed that the nanoparticles were found to be negatively charged, which implied that the particles were stable (Figure 6) [27].

### 3.2. Toxicity Studies on Earthworm and Zebrafish

#### 3.2.1. Phenotypical Changes

**Figure 7:**
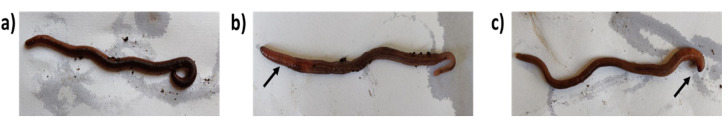
Phenotypic changes observed in earthworms exposed to silver nanoparticles. (**a**) control; (**b**) exposed to 10 µg; (**c**) exposed to 20 µg (Black arrow indicates the brownish pink changes).

**Figure 8:**
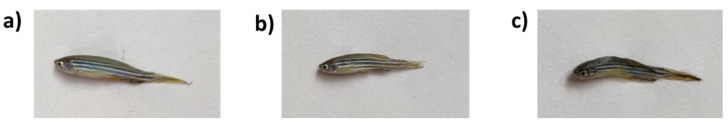
Phenotypic changes observed in zebrafish exposed to silver nanoparticles. (**a**) control; (**b**) exposed to 10 µg; (**c**) exposed to 20 µg.

The earthworms showed change in color from being brownish black to brownish pink (Figure 7). But there were no noticeable changes in behavior and body mass (Table 1), unlike many other studies which have been reported on earthworms being slow, with sluggish movement, and with much morphological changes [29]. The exposure to silver nanoparticles affected its reproductive and death rate (Table 2). Samrot et al. [30] reported on the color changes in earthworms caused by nanoparticles and confirmed that nanoparticles can enter inside the epithelium easily. Silver nanoparticles tend to impart a negative effect on the rate of reproduction of earthworm [31]. The phenotypical changes of zebrafish exposed to silver nanoparticles are tabulated in Table 3 (Figure 8). Zebrafishes exposed to a lower concentration of 10 µg showed slight changes in appearance and behavior due to metal nanoparticle deposition. The gills and fins of fishes exposed to 20 µg of nanoparticles exhibited a yellowish tint. The fishes were found to be weak and slow, and their growth was inhibited. Based on the results of the experiments, silver nanoparticle exposure caused phenotypical and mortality changes that were concentration–dependent.

#### 3.2.2. Inductively Coupled Plasma Optical Emission Spectroscopy (ICP–OES)

**Figure 9:**
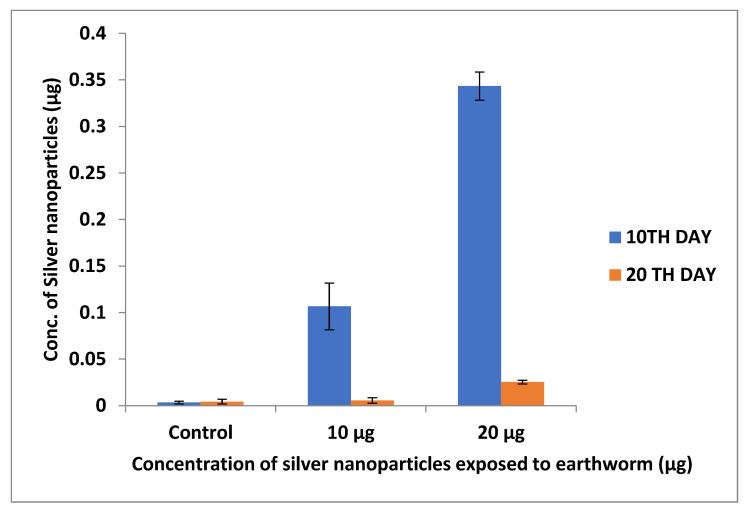
Silver nanoparticle accumulation study using ICP–OES in earthworms.

**Figure 10:**
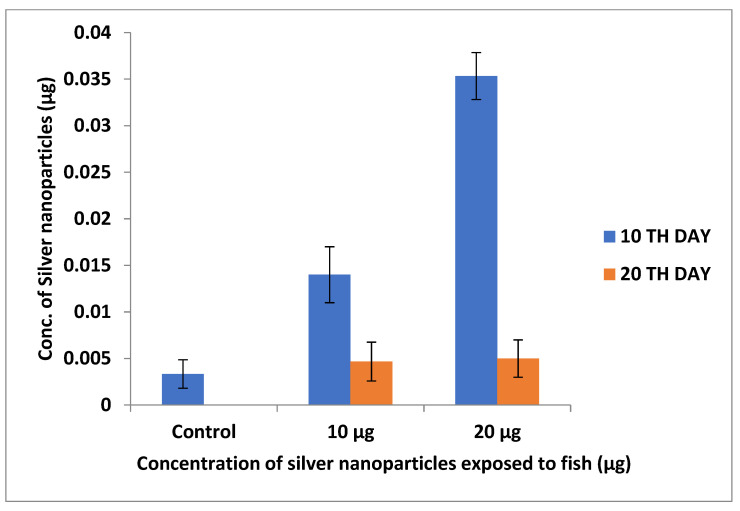
Silver nanoparticle accumulation study using ICP–OES in zebrafishes.

The silver deposition in earthworms and zebrafishes was investigated using ICP–OES analysis. The accumulation was observed to be greater at 20 µg concentrations due to the increased concentration of nanoparticles. Samrot et al. [22] reported that SPIONs accumulation increases with an increase in nanoparticle concentration in treated earthworms (Figure 9 and Figure 10).

#### 3.2.3. Histology Studies

**Figure 11:**
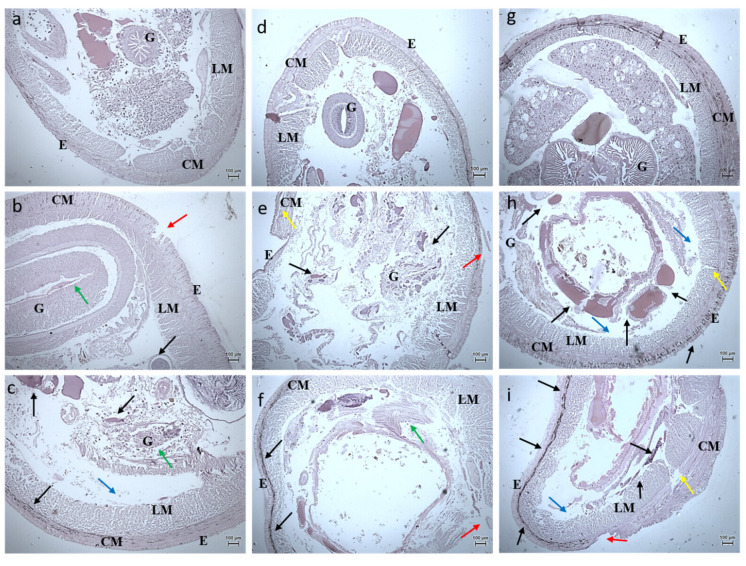
Histology images of foregut region of earthworm—*Eudrilus eugeniae* (10X Magnification) (**a**) 10th day control, (**b**) exposed to 10 µg at 10th day, (**c**) exposed to 20 µg at 10th day; (**d**) 20th day control, (**e**) exposed to 10 µg at 20th day, (**f**) exposed to 20 µg at 20th day; (**g**) 30th day control, (**h**) exposed to 10 µg at 30th day, (**i**) exposed to 20 µg at 30th day. G—Gut, E—Epidermis, LM—Longitudinal Muscle, CM—Circular Muscle. Black arrow denotes lipofuscin, red arrow denotes erosion of epidermis, blue arrow denotes interstitial space, yellow arrow denotes fibrosis of CM, green denotes arrow disruption of gut.

**Figure 12:**
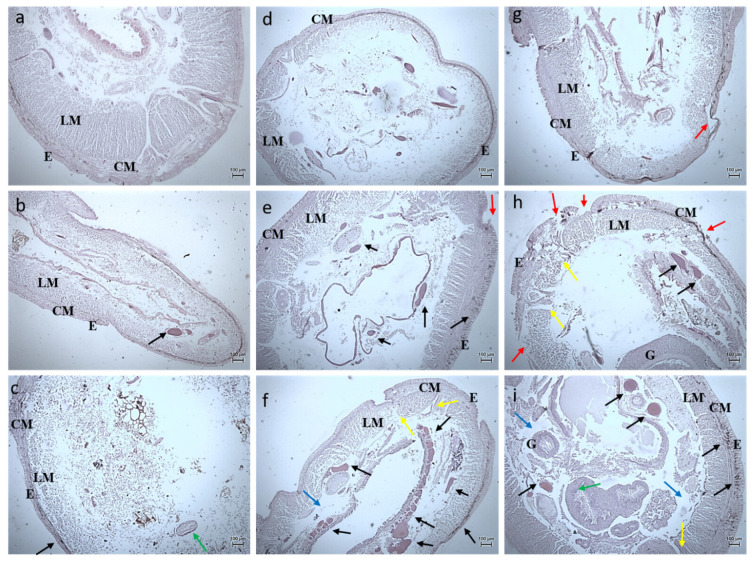
Histology images of midgut region of earthworm—*Eudrilus eugeniae* (10X Magnification) (**a**) 10th day control, (**b**) exposed to 10 µg at 10th day, (**c**) exposed to 20 µg at 10th day; (**d**) 20th day control, (**e**) exposed to 10 µg at 20th day, (**f**) exposed to 20 µg at 20th day; (**g**) 30th day control, (**h**) exposed to 10 µg at 30th day, (**i**) exposed to 20 µg at 30th day. G—Gut, E—Epidermis, LM—Longitudinal Muscle, CM—Circular Muscle. Black arrow denotes lipofuscin, red arrow denotes erosion of epidermis, blue arrow denotes interstitial space, yellow arrow denotes fibrosis of CM, green denotes arrow disruption of gut.

**Figure 13:**
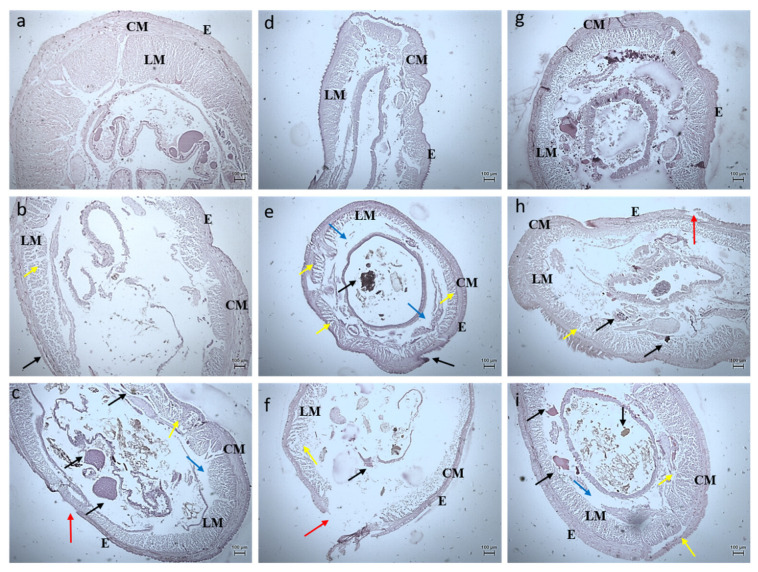
Histology images of hindgut region of earthworm—*Eudrilus eugeniae* (10X Magnification) (**a**) 10th day control, (**b**) exposed to 10 µg at 10th day, (**c**) exposed to 20 µg at 10th day; (**d**) 20th day control, (**e**) exposed to 10 µg at 20th day, (**f**) exposed to 20 µg at 20th day; (**g**) 30th day control, (**h**) exposed to 10 µg at 30th day, (**i**) exposed to 20 µg at 30th day. E—Epidermis, LM—Longitudinal Muscle, CM—Circular Muscle. Black arrow denotes lipofuscin, red arrow denotes erosion of epidermis, blue arrow denotes interstitial space, yellow arrow denotes fibrosis of CM.

**Figure 14:**
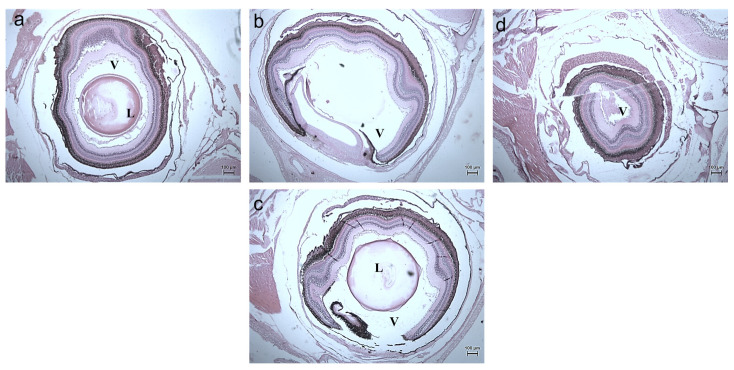
Histology images of eyes of Zebrafish—*Danio rerio* (10X Magnification) (**a**) 10th day Control, (**b**) 20th day Control, (**c**) exposed to 10 µg at 20th day, (**d**) 30th day Control. L–Lens, V–Vitreous.

**Figure 15:**
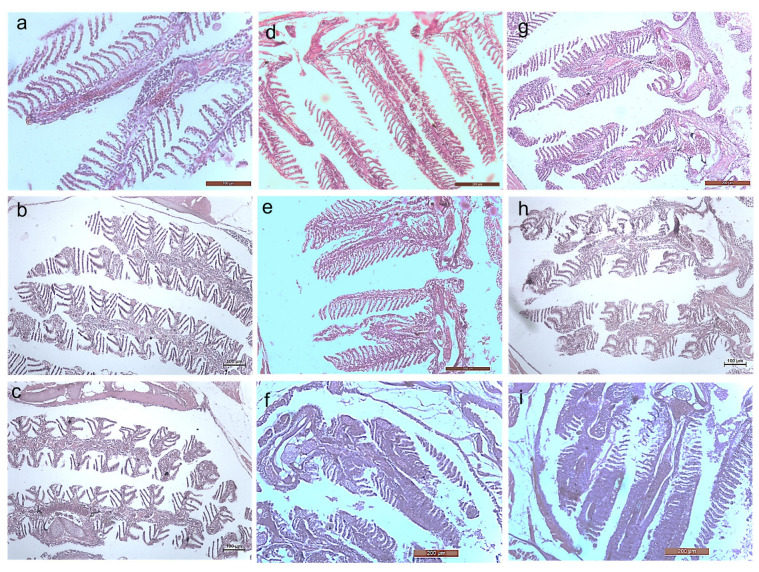
Histology images of gills of Zebrafish—*Danio rerio* (10X Magnification) (**a**) 10th day control, (**b**) exposed to 10 µg at 10th day, (**c**) exposed to 20 µg at 10th day; (**d**) 20th day control, (**e**) exposed to 10 µg at 20th day, (**f**) exposed to 20 µg at 20th day; (**g**) 30th day control, (**h**) exposed to 10 µg at 30th day, (**i**) exposed to 20 µg at 30th day.

**Figure 16:**
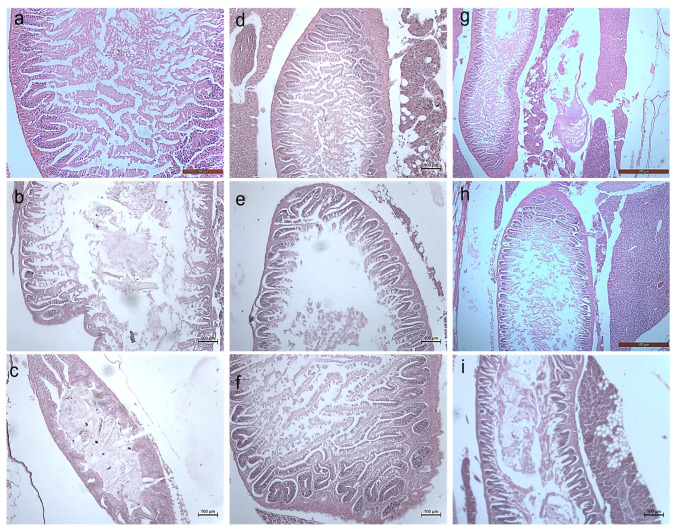
Histology images of intestines of Zebrafish—*Danio rerio* (10X Magnification) (**a**) 10th day control, (**b**) exposed to 10 µg at 10th day, (**c**) exposed to 20 µg at 10th day; (**d**) 20th day control, (**e**) exposed to 10 µg at 20th day, (**f**) exposed to 20 µg at 20th day; (**g**) 30th day control, (**h**) exposed to 10 µg at 30th day, (**i**) exposed to 20 µg at 30th day.

**Figure 17:**
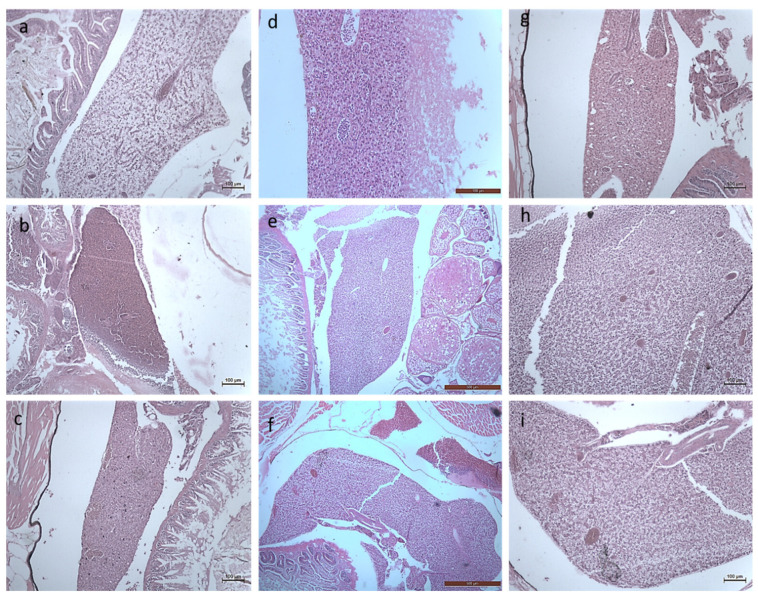
Histology images of liver of Zebrafish—*Danio rerio* (10X Magnification) (**a**) 10th day control, (**b**) exposed to 10 µg at 10th day, (**c**) exposed to 20 µg at 10th day; (**d**) 20th day control, (**e**) exposed to 10 µg at 20th day, (**f**) exposed to 20 µg at 20th day; (**g**) 30th day control, (**h**) exposed to 10 µg at 30th day, (**i**) exposed to 20 µg at 30th day.

The toxicity of the synthesized *Bauhinia purpurea* gum mediated silver nanoparticles were examined through histological images of the earthworm and zebrafishes exposed to two different concentrations (Figure 11, Figure 12, Figure 13, Figure 14, Figure 15, Figure 16 and Figure 17).

Head, mid, and the tail region of the earthworm were observed for possible toxicity. In the head region, no changes at a lower concentration were noticed. At higher concentrations, the epidermis and the underlying circular muscles were eroded, and the gizzard region was found to be disintegrated. On 20th day, fibrosis in the circular muscles and lipofuscin—like granules were observed with epidermis deformation in the earthworm exposed to 20 µg. Samrot et al. [12] reported that increasing the concentration of silver nanoparticles caused fibrosis in the circular muscle. Exposure to silver nanoparticles tends to induce erosion and fibrosis in *Lumbricus rubellus* earthworms due to the concentration-dependent accumulation of nanoparticles in the gut region and tissue [32]. Bourdineaud et al. [33] imparted an increase in the level of MDA (Malondialdehyde) causing oxidative stress in earthworms on exposure to gold nanoparticles.

On exposure to nanoparticles, there was erosion in the sclera, the photoreceptors, and cornea of the zebrafishes treated with a lower concentration of silver nanoparticles (Figure 14b,e,h). At a higher concentration, the vitreous humor, lens, iris, and suspensory ligament were eroded completely (Figure 14c,f,i) compared to the control (Figure 17a,d,g). The gills of the fish were found to be damaged heavily and at a higher concentration, the gill rakers, arches, and filaments were also damaged (Figure 12). At a lower concentration, no damages in the intestine were seen. The deformation in the intestinal walls and erosion of goblet cells in the intestinal epithelium of fishes exposed to a higher concentration were observed (Figure 16). The degradation of the hepatocytes in the liver leading to the vascular degeneration in the fish exposed to silver nanoparticles were also observed (Figure 17), in addition to the erosion of sinusoids in the liver at a higher concentration of exposure. Charged gold nanoparticles have the ability to cause inflammatory and altered immune effects on zebrafish [34]. Griffitt et al. [35] reported that copper nanoparticles were found to be extremely toxic to zebrafishes, causing damage in the gill lamellae and skin. Modulation of cytokines caused by metal oxide nanoparticles by the generation of free radicals has also been reported [36]. The alteration in the genomic composition has been detected in fishes treated even with a lesser concentration [37]. Sheng et al. [38] reported on the suppression in the genes, such as p38, CRE, NGF, which has led to brain damage causing neurotoxicity.

**Figure 18:**
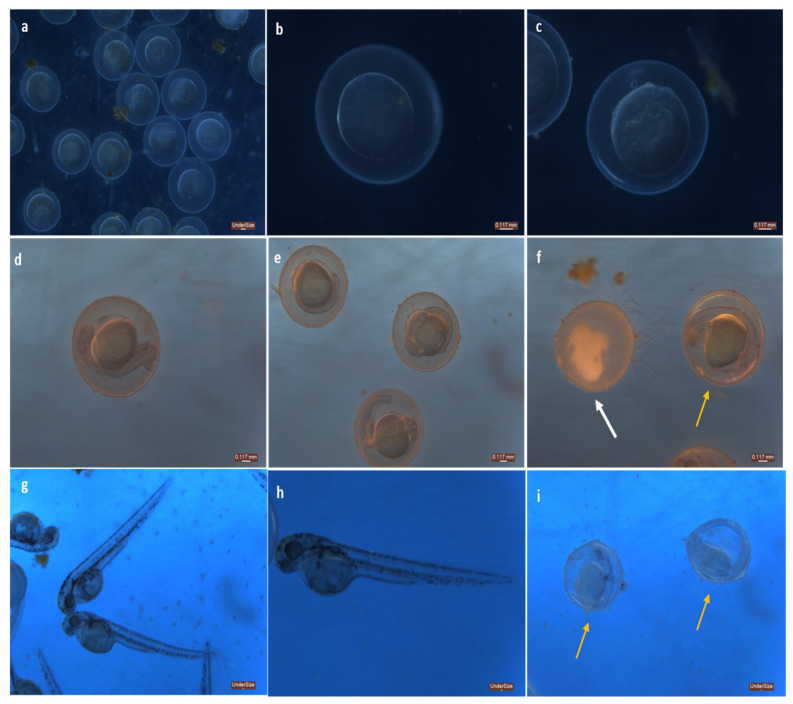
Microscopic images of Zebrafish—*Danio rerio* embryos (**a**) control at 0th h, (**b**) exposed to 10 µg at 0th h, (**c**) exposed to 20 µg at 0th h, (**d**) control at 24 hpf, (**e**) exposed to 10 µg at 24 hpf, (**f**) exposed to 20 µg at 24 hpf, (**g**) control at 48 hpf, (**h**) exposed to 10 µg at 48 hpf, (**i**) exposed to 20 µg at 48 hpf. White arrow denotes dead embryos (coagulation); Yellow arrows denote undeveloped embryos.

The zebrafish embryos were treated with silver nanoparticles of two different concentrations—10 µg and 20 µg (Figure 18).

The embryos were found to be unaffected on exposure to nanoparticles at the 0th h. At 24 h post—fertilization (hpf), deformation in the chorion and cerebellum was seen at lower concentrations whereas at higher concentration, coagulated eggs with intransparent inclusions were observed (Table 4 and Table 5). At 48 hpf, hatching of embryos to form larvae was higher compared to exposure at a higher concentration, indicating that it had an impact on the hatching rate of the embryos and delayed the process of embryogenesis. The embryos were found to be underdeveloped and slightly deformed. According to Xia et al. [39], lower concentrations of silver nanoparticles had no effect on zebrafish embryos, however greater concentrations caused damage to the mesodermal and ectodermal tissues. TMAT–functionalized gold nanoparticles of 1.3 nm size caused improper eye development, including alterations in pigmentation and neuronal damage, as well as behavioral problems [40]. Pericardial oedema, bent tail malformation, spinal curvature, non-inflated swim bladder, and yolk–sac oedema are some of the most commonly found defects in zebrafish embryos [41,42]. Silver nanoparticles and silver nitrate were investigated for toxicity against the freshwater fish *Oreochromis mossambicus*. It has been shown that both induced telangiectasia and epithelial cell hyperplasia, as well as silver accumulation which caused ineffective oxidative stress and altered enzymatic and non-enzymatic parameters, resulting in cellular damages [43]. Exposure to chemically synthesized silver nanoparticles can affect the rate of DNA replication and the cellular senescence pathway. It can disrupt the mitochondrial respiratory chain and influence DNA replication, cellular senescence, and mitochondrial respiratory chain—related genes at the transcriptional level [44].

## 4. Conclusions

In this study, silver nanoparticles were synthesized using plant gum of *Bauhinia purpurea* as the reducing agent, and the toxicity of silver nanoparticles was investigated against earthworms, *Danio rerio*, and its embryos. The nanoparticles were crystalline and had sizes ranging between 13 and 20 nm. These biologically produced nanoparticles showed less impact on the rate of reproduction but showed increased mortality on earthworms as the concentration increased. It was found to be less toxic on embryos and various organs of *Danio rerio*. Thus, this work suggests that the silver nanoparticles synthesized using biogenic molecules are less toxic than the ones synthesized using a chemical method and needs to be disposed properly to minimize negative consequences to the environment.

## Figures and Tables

**Table 1:** Phenotypic changes observed in earthworms exposed to silver nanoparticles synthesized using *Bauhinia purpurea* gum.

Parameter Analyzed	Control		10 µg		20 µg	
	Before	After	Before	After	Before	After
Color change	Brownish black	Brownish black	Brownish black	Brownish pink	Brownish black	Brownish pink
Behavioral change	Nil	Nil	Nil	Nil	Nil	Nil

**Table 2:** Total count of earthworms exposed to silver nanoparticles synthesized using *Bauhinia purpurea* gum.

Conc.	Before	10th day	20th day	30th day
Control	10	22	27	51
10 µg	10	17	22	14
20 µg	10	17	13	9

**Table 3:** Phenotypic changes observed in zebrafish exposed to silver nanoparticles synthesized using *Bauhinia purpurea* gum.

Parameter Analyzed	Control		10 µg		20 µg	
	Before	After	Before	After	Before	After
Color change	Nil	Nil	Nil	Yellowish tint in the gills and fins	Nil	Yellowish tint in the gills and fins
Behavioral change	Active	Active	Active	Weak and slow	Active	Weak and slow
Growth	Normal	Normal	Normal	Inhibition in growth	Normal	Inhibition in growth

**Table 4:** Total count of zebrafishes exposed to silver nanoparticles synthesized using *Bauhinia purpurea* gum.

Conc.	Before	10th Day	20th Day	30th Day
Control	10	10	10	10
10 µg	10	10	9	9
20 µg	10	9	8	8

**Table 5:** Apical observations observed for zebrafish embryos.

	Control			10 µg			20 µg		
	0th h	24 hpf	48 hpf	0th h	24 hpf	48 hpf	0th h	24 hpf	48 hpf
Hatching	NA	NA	+++	NA	NA	++	NA	NA	+
Coagulated embryos	-	+	-	-	+	++	-	+	++
Somite formation	+	+	+	+	+	+	+	+	+
Heartbeat	NA	NA	++	NA	NA	++	NA	NA	+

NA—Non applicable, + denotes good, ++ denotes better, +++ denotes best, - denotes negative result.

## Data Availability

The data used to support the findings of this study are included in the article. Should further data or information be required, these are available from the corresponding author upon request.
